# Unmet needs in health training among nurses in rural Chinese township health centers: a cross-sectional hospital-based study

**DOI:** 10.3352/jeehp.2017.14.22

**Published:** 2017-10-04

**Authors:** Yan Mo, Guijie Hu, Yanhua Yi, Yanping Ying, Huiqiao Huang, Zhongxian Huang, Jiafeng Lin

**Affiliations:** 1 First Affiliated Hospital of Guangxi Medical University, Guangxi, China; 2 The Maternal and Child Hospital of Guangxi Zhuang Autonomous Region, Guangxi, China; 3 Department of Plastic and Aesthetic Surgery, Nan Fang Hospital, Southern Medical University, Guangdong, China; Hallym University, Korea

**Keywords:** China, Health training, Nurse, Human resources for health, Rural health care, Township health centers

## Abstract

**Purpose:**

Maintaining a sufficient and competent rural nursing workforce is an important goal of the Chinese health delivery system. However, few studies have investigated the health training status or conducted a needs assessment of rural Chinese nurses during this time of great transformations in health policy. This study was conducted to explore the current health training status of nurses working in rural Chinese township health centers (THCs) and to ascertain their perceived needs.

**Methods:**

A cross-sectional survey using a self-administered structured questionnaire was conducted among 240 THC nurses in Guangxi Zhuang Autonomous Region, China from March 2014 to August 2014. The survey questionnaire was adapted from the Second Chinese Survey of Demographic Data and Training Demand for Health Professionals in THCs developed by the Ministry of Education.

**Results:**

The nurses in THCs were young, with a low educational level. Their perceived needs for health training included further clinical studies at city-level hospitals to improve their skills and theoretical studies at medical universities in emergency medicine and general practice. Overall, 71.9% of the nurses with a secondary technical school background expected to pursue junior college studies, and 68.5% of the nurses with a junior college education expected to pursue a bachelor’s degree. A decentralized program with theoretical studies at medical universities and practical studies at county hospitals was regarded as feasible by 66.9% of the respondents.

**Conclusion:**

Health-training programs for nurses in Chinese THCs must be improved in terms of coverage, delivery mode, and content. A decentralized degree-linked training program in which medical universities and city hospitals collaborate would be an appropriate mode of delivery.

## Introduction

Since 1993, the Chinese Ministry of Health (MoH) has annually administered a national nursing licensing examination and has required nurses to obtain a nursing practice certificate. The first Nurse Act was issued by the Chinese government in 2008. The Act specified that a minimum of 3 years of full-time study (including 8 months of clinical placement) in a nursing program accredited by the Ministry of Education (MoE) or the MoH was required to take the nursing licensing examination [1]. Currently, several paths lead to eligibility to become a registered nurse, including secondary, junior college, and undergraduate programs. Far too few nurses graduate from undergraduate programs at medical universities to serve all the hospitals in big cities. Within the 3-tier health service system in rural China, township health centers (THCs) link county hospitals at the third tier and village clinics at the first tier, and must recruit nurses from secondary or junior college nursing programs [2].

It has been documented that health training programs play a significant role in enabling THC health providers to improve the quality of their services and help increase rural retention [3]. Currently, there are 2 kinds of health training programs available for nurses in China. The first is a degree-linked adult education program. Nurses can upgrade their degree by attending courses at medical universities and receive degrees conferred by the provincial department of education. The second type consists of non-degree health training programs, in which nurses can enroll in further clinical studies in higher-level hospitals or in conference sessions organized by local nurses’ associations. The Chinese MoE issued “the training provisions for health workers in township health centers” in 2004, which required that the new health workers working in THCs should have at least 1 year of training in county- or city-level hospitals to learn clinical skills after they graduate from medical school, and that they should receive more than 3 months of clinical training after 5 years of work in THCs [4]. These provisions also encouraged nurses who have obtained nursing licenses to enroll in degree studies to upgrade their education level. To date, little is known regarding the current status of health training among rural nurses in THCs and their perceived needs.

Of the total population of 51.59 million in Guangxi Zhuang Autonomous Region (hereafter abbreviated as Guangxi), a province in southwestern China, 82% live in rural areas [5]. The rate of nurses per 1,000 rural population in Guangxi was 0.38 in 2013. There are 1,279 THCs in Guangxi [6]. A shortage of competent nursing health workers has been a challenge for the rural health system for many years. Health training promotes nurses’ professional development, helping them to gain more confidence in serving rural inhabitants, which will ultimately increase retention. In the past decade, THCs have begun to play more active roles in public health and preventive medicine; some nurses in THCs have transferred from clinical service to public health, or even to hospital management. New health training programs should be planned that cater to the new needs of nurses in THCs.

Various training programs have been implemented for nurses in THCs in recent years. These programs include conference sessions, further clinical education in county or city hospitals, studying in medical schools, guidance from senior colleagues within the THC that they work in (case studies), self-education, and remote video training. Almost all these programs are organized and regulated by hospitals or other healthcare organizations, and the needs of learners have therefore rarely been assessed. Currently, 11 years after the Chinese MoE issued training provisions for THC health workers, it is imperative to identify whether the training programs have included all nurses, and which delivery modes, content, and settings for training should be implemented. This study aimed to investigate the current health training status of nurses in THCs and their perceived needs.

## Methods

### Study design

**Fig. 1.**
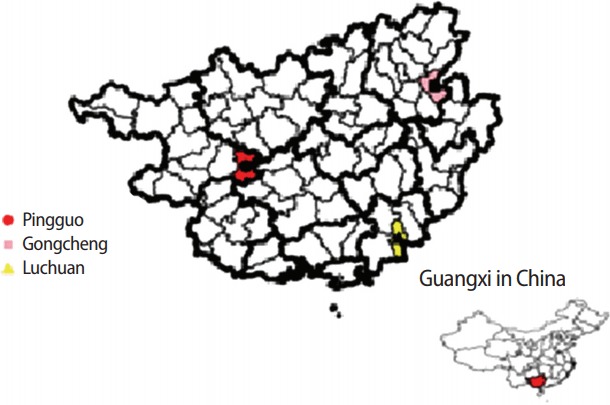
Counties sampled in Guangxi.

A self-administered questionnaire was used in this cross-sectional survey conducted in Pingguo, Gongcheng, and Luchuan, 3 counties located in the northwest, northeast, and southeast of Guangxi, respectively (Fig. 1). The counties in Guangxi have been categorized according to socioeconomic development indices into high, middle, and low levels [5]. Pingguo, Gongcheng, and Luchuan are representative counties of the 3 levels, randomly selected from the counties with high, middle, and low socioeconomic levels of development, respectively.

### Study population

There were 279 nurses in the 3 counties. After the exclusion of 39 nurses who did not have a nursing license because they had been working for less than half a year or who were planning to retire in the coming year, 240 nurses from the 3 counties with nursing licenses who were practicing in all the THCs were included.

### Data collection method and instruments

Data collection was carried out from March 2014 to August 2014. Based on a literature review and interviews, for this survey, we adapted the questionnaire that was used in the second Chinese survey of demographic data and training demand for health professionals in THCs by the MoE in 2011 [7]. To ensure its validity, a statistician, 2 epidemiologists, 4 health human resources managers, and 10 nurses working in THCs reviewed the questionnaires. A small pilot study was conducted in the Daqiao township hospital in Luchuan County to verify the suitability of the content of the questionnaire.

### Data analysis

The data were entered into EpiData software 3.0 version (EpiData Association, Odense, Denmark) and cross-verified for consistency. R version 3.3.1 (The R Foundation for Statistical Computing, Vienna, Austria) [8] and the epicalc package ver. 2.15.1.0 (The R Foundation for Statistical Computing) [9] were used for data analysis. For categorical variables, the chi-square test or the Fisher test was used as appropriate. Statistical significance was set at 5%.

### Ethical approval

The Ethics Committee of Guangxi Medical University approved the study protocol (No: GXMU-IRB-2014-116) after receiving written informed consent from the subjects.

## Results

### Demographic information

Of the 240 working nurses recruited in the study, 233 (97.1%) participated and completed the questionnaire. Table 1 summarizes the demographic information of the respondents. They were young (73.4% were younger than 35 years old) with a short duration of work experience (median= 8 years) in THCs. The majority of respondents had a low level of formal education. Pingguo and Gongcheng had larger percentages of nurses with junior college degrees than Luchuan. Three levels of technical titles (junior, intermediate, and senior) are used to indicate nurses’ competency and seniority based on examinations and reviews from the health department. Only 15.5% of respondents had intermediate technical titles, the majority (84.5%) had no or junior technical titles, and none had senior titles. Raw data were available from Supplement 1.

### Mismatch between current health training initiatives and desired programs in terms of training setting, content, and delivery mode

As summarized in Table 2, 40.2% and 37.3% of nurses reported that they would like to enroll in health training at medical colleges and city hospitals, respectively, but the current initiatives only provided training at medical colleges to 6.8% of respondents and training at city hospitals to 6.8% of respondents. Although 43.2% responded that they had received some form of health training in the THC where they currently worked, only 3.9% of them intended to enroll in such training in the future. Emergency medicine and general practice knowledge were perceived to be needed by 58.8% and 27% of respondents, respectively, but only 39.1% and 10.3% of the respondents had attended such trainings. Of the respondents, 60.1% and 52.8% had attended training initiatives focused on basic theory and clinical skills, respectively, but only 18% and 36.9% of these nurses intended to attend such trainings in the future. Moreover, 43.4% of respondents intended to enroll in further clinical studies (the most desirable delivery mode), but only 10.9% of them had received the chance to do so.

### Needs assessment for educational degree studies

Table 3 shows that 71.9% of the nurses with a secondary technical school background and 68.5% of nurses with a junior college education expected to enroll in junior college and bachelor’s degree studies, respectively. Part-time learning combining theoretical studies at medical universities and practical studies at city hospitals was perceived as the most ideal learning program. More than half of the nurses could afford 1,000–1,500 renminbi (161.3–242.1 US dollar) for degree studies every year. A decentralized program with theoretical studies at medical universities and practical studies at county hospitals was regarded as feasible by 66.9% of the respondents.

## Discussion

The findings that the nurses in THCs were young with a short duration of work experience, a low level of formal education, and no or junior technical titles have important implications for health training planners. Although the nursing community has reached a consensus that the secondary nursing programs should be phased out gradually, 862 secondary nursing schools are still producing large number of nurses for THCs. Due to their youth and low educational level, these nurses require further health training to be sufficiently prepared for the growing demands for rural health services [10]. Therefore, investment in appropriate health training for these young nurses would have a lasting return for rural primary health services [11].

Above all, the government should take prompt measures to provide health training programs to all THC nurses according to “the training provisions for health workers in township health centers.” In addition, health training planners should be aware of the remarkable mismatch between current health training initiatives and desired programs in terms of delivery mode, content, and the training setting. The young nurses included in this study would like to attend training programs at medical universities to improve their knowledge of medical theory, and to pursue further clinical studies at higher-level hospitals to improve their clinical competency. Moreover, the majority intended to upgrade their educational degrees through part-time degree-linked programs. If medical universities cooperate with city or county hospitals to create a decentralized degree-linked health training program, focusing on emergency medicine and general practice, this would take the nurses’ perceived needs into account and result in a better learner-centered health training program. This study confirmed that more than two-thirds of respondents regarded a decentralized program combining theoretical studies at medical universities and practical studies at county hospitals as a feasible training program. A decentralized program has been proven to be effective and popular in other countries for nurses and other health workers [12,13]. It would be appropriate for nurses in THCs.

This study also reflected the fact that urbanization in China has affected the content perceived to be necessary in health training. Rapid urbanization with convenient transportation has allowed rural residents to seek out medical services for serious diseases in large cities. The scope of rural medical services has generally included dealing with emergent cases and maintaining the availability of essential drugs for non-communicable diseases, which is why the need for health training in emergency medicine and general practice was perceived so widely. In addition, urbanization has facilitated the mobility of nurses to higher-level hospitals or nonmedical professions [14]. In 2010, the Chinese government launched “the rural-oriented tuitionwaived medical education program.” This program was designed to recruit students from rural towns to study at medical universities for 5 years. The government waived students’ tuition and gave them a monthly stipend. The students were required to sign a contract with a local THC obliging them to serve in a THC for at least 6 years after graduation. This ‘contract training mode’ could also be applied to health training for nurses working in THCs. This program is expected to foster future nursing leadership in THCs, and to improve retention. The fact that most nurses preferred to study in city-level hospitals instead of learning from senior colleagues in the THCs where they worked also reflected poor faculty development in rural health training.

Within the context of central government planning, the situation is more or less the same in most areas of rural China, especially in western underdeveloped regions. These findings have implications for health training planners in China, as well as in other countries with similar circumstances. However, due to limitations of budget and time, this study could not include many other stakeholders in health training. A stakeholder analysis to clarify the feasibility of a decentralized degree-linked training program for nurses in THCs would be essential.

In conclusion, health-training programs for nurses in Chinese THCs must be improved in terms of coverage, delivery mode, and content. A decentralized degree-linked training program in which medical universities and city hospitals collaborate would be an appropriate mode of delivery.

## Figures and Tables

**Table 1.** Demographic information of the nurses from 3 counties in Guangxi

Demographic	Category	Pingguo (n = 61)	Gongcheng (n = 43)	Luchuan (n = 129)	Total (n = 233)
Age (yr)	< 25	14 (23.0)	8 (18.6)	36 (27.9)	58 (24.9)
	25–34	29 (47.5)	24 (55.8)	60 (46.5)	113 (48.5)
	35–44	15 (24.6)	6 (14.0)	27 (20.9)	48 (20.6)
	≥ 45	3 (4.9)	5 (11.6)	6 (4.7)	14 (6.0)
Working position	Nursing	47 (81.0)	34 (82.9)	112 (88.9)	193 (85.8)
	Public health	10 (17.2)	6 (14.6)	14 (11.1)	30 (13.3)
	Administration	1 (1.7)	1 (2.4)	0 (0.0)	2 (0.9)
Work experience (yr)	Median (interquartile range)	8 (4.0–13.2)	10 (6.5–14.0)	8 (4.0–14.0)	8 (4.0–14.0)
Education^***^	Senior secondary school and below	1 (1.6)	1 (2.3)	2 (1.6)	4 (1.7)
	Secondary technical school	19 (31.1)	13 (30.2)	78 (60.5)	110 (47.2)
	Junior college	38 (62.3)	29 (67.4)	49 (38.0)	116 (49.8)
	Undergraduate	3 (4.9)	0 (0.0)	0 (0.0)	3 (1.3)
Technical titles	No title	26 (43.3)	18 (41.9)	73 (56.6)	117 (50.2)
	Junior	21 (35.0)	17 (39.5)	42 (32.6)	80 (34.3)
	Intermediate	13 (21.7)	8 (18.6)	14 (10.9)	35 (15.5)

Values are presented as number (%).

*** P<0.001.

**Table 2.** Current health training initiatives versus desired training programs in terms of setting, content of training, and delivery mode

Variable	Category	Current initiatives (%)	Desired programs (%)
Place of training	Medical colleges	6.8	40.2
	City hospital	11.1	37.3
	County hospital	38.3	18.6
	Current workplace	43.2	3.9
Content of training^a)^	Preventive health	36.1	34.8
	Rational drug use	15.5	5.2
	Clinical methodology	9.0	8.6
	Emergency medicine	39.1	58.8
	General practice knowledge	10.3	27.0
	Locally appropriate technology	16.3	29.6
	Clinical skills	52.8	36.9
	Basic theory	60.1	18.0
Delivery modes	Remote video education	2.5	1.1
	Self-education	13.0	8.2
	Guidance from senior colleagues	42.8	18.7
	Study in medical schools	8.4	8.2
	Further clinical education	10.9	43.4
	Conference sessions	22.5	20.3

a) Indicates multiple options.

**Table 3.** Factors related to intentions to pursue junior college and bachelor’s degree studies

Variable	Junior college (n = 64)	Bachelor’s degree (n = 108)	Total (n = 172)
Age (yr)			
< 25	20 (31.2)	27 (25.0)	47 (27.3)
25–44	39 (60.9)	79 (73.1)	118 (68.6)
≥ 45	5 (7.8)	2 (1.9)	7 (4.1)
Education^***^			
Senior secondary school and below	2 (3.1)	0 (0.0)	2 (1.2)
Secondary technical school	46 (71.9)	34 (31.5)	80 (46.5)
Junior college	16 (25.0)	74 (68.5)	90 (52.3)
Preferred types of study			
Part-time learning	31 (50.8)	51 (48.1)	82 (49.1)
Full-time learning	14 (23.0)	40 (37.7)	54 (32.3)
Self-study	16 (26.2)	15 (14.2)	31 (18.6)
Preferred method for theoretical studies			
Video teaching	22 (35.5)	36 (34.6)	58 (34.9)
Adult education at medical universities	35 (56.5)	66 (63.5)	101 (60.8)
Self-study	5 (8.1)	2 (1.9)	7 (4.2)
Preferred place for practical studies^*^			
Present workplace	3 (4.9)	7 (6.5)	10 (6.0)
County hospital	20 (32.8)	26 (24.3)	46 (27.4)
City hospital	36 (59.0)	52 (48.6)	88 (52.4)
College teaching hospitals	2 (3.3)	22 (20.6)	24 (14.3)
Affordable annual fees for studies (renminbi)			
1,000–1,500	36 (59.0)	63 (58.9)	99 (58.9)
1,500–2,000	13 (21.3)	24 (22.4)	37 (22.0)
2,000–2,500	11 (18.0)	15 (14.0)	26 (15.5)
> 2,500	1 (1.6)	5 (4.7)	6 (3.6)
Feasibility of a decentralized program^a)^			
Very feasible	15 (24.6)	23 (22.5)	38 (23.3)
Feasible	29 (47.5)	42 (41.2)	71 (43.6)
Neutral	16 (26.2)	29 (28.4)	45 (27.6)
Not feasible	1 (1.6)	5 (4.9)	6(3.7)
Absolutely not feasible	0	3 (2.9)	3 (1.8)

Values are presented as number (%).

* P<0.05.

*** P<0.001.

a) Refers to a decentralized program combining theoretical studies at medical universities and practical studies at county hospitals.

## Supplementary materials

Supplement 1. Data file is available from https://doi.org/10.7910/DVN/IXBF1L.

Supplement 2. Audio recording of the abstract.
